# Unveiling the diversity and mechanisms of plant growth-promoting bacteria in orchids: a comprehensive review

**DOI:** 10.3389/fmicb.2026.1697953

**Published:** 2026-01-29

**Authors:** Faiza Ramzan, Loukia Vassiliou, Dimitrios Tsaltas

**Affiliations:** 1 Department of Agricultural Sciences, Biotechnology and Food Science, Cyprus University of Technology, Limassol, Cyprus; 2 Agricultural Research Institute, Nicosia, Cyprus

**Keywords:** biocontrol, IAA production, metagenomics, nitrogen fixation, Orchidaceae, PGPB, symbiotic interactions

## Abstract

Orchids, one of the most diverse and ecologically important plant families, form complex associations with endophytic microorganisms that are vital for their survival, growth, and adaptation. These endophytes, including both fungi and bacteria, inhabit orchid tissues without causing harm and contribute to key physiological processes such as nutrient acquisition, stress tolerance, and disease resistance. This review explores the diversity and ecological roles of orchid-associated endophytes, emphasizing their significance in promoting germination, biomass production, and resilience to environmental stressors. Plant Growth-Promoting Bacteria (PGPB) such as *Pseudomonas*, *Bacillus*, and *Burkholderia* enhance nutrient uptake and plant defense, offering eco-friendly alternatives to chemical fertilizers and pesticides. Beyond ecological functions, endophytes show potential in biotechnology for sustainable agriculture, conservation, and novel bioactive compound discovery. Despite advances in molecular tools like metagenomics and next-generation sequencing, challenges persist in fully understanding and utilizing these microbes. This review highlights the need for multidisciplinary collaboration to optimize microbial inoculants, elucidate symbiotic mechanisms, and develop practical applications for conservation and sustainable horticulture. By integrating fundamental research with applied strategies, this work aims to unlock the full potential of orchid-associated endophytes in ecological and commercial domains.

## 1 Introduction

Orchids, with their stunning beauty and delicate appearance, have captured the hearts of botanists and garden enthusiasts for centuries. These unique and diverse plants belong to one of the largest families of flowering plants, with approximately 30,000–35,000 species distributed worldwide. They have successfully colonized every habitat on earth, ranging from tropical rainforests to high elevation areas and even extreme conditions like desert, savannas and arctic tundra. The success of orchids in such diverse environments can be attributed to a variety of factors, like symbiotic relationships, soil pH, water content in the soil, temperature, etc.

Orchids form a unique species-specific symbiotic association with mycorrhizal fungi, which is essential during the early stages of development, particularly seed germination. Due to their tiny, endosperm-lacking seeds, orchids depend on these fungi for nutrient and water uptake, making mycorrhizal interactions critical for successful germination and overall life cycle progression (Miura et al., 2023; Pujasatria et al., 2024; Zhao et al., 2024). Although this fungal association has traditionally been viewed as the central component of orchid biology, it represents only one facet of a much broader microbial network associated with orchids.

Beyond fungi, orchids interact with a diverse community of microorganisms inhabiting both the endosphere and rhizosphere, many of which significantly influence germination, nutrient acquisition, growth, and stress resilience (Kaur et al., 2023; Kumar Tamrakar and Singh Parihar, 2023; Kumar et al., 2023). Mycorrhiza has been investigated in detail in terms of taxonomy, specificity, and interaction with orchid plants, but very limited information is available on the other microorganisms like bacteria despite growing evidence of their ecological importance (Li et al., 2021). These understudied bacterial communities play crucial roles in plant health, often acting independently or synergistically with fungi to enhance orchid performance across developmental stages.

The rhizosphere, a narrow soil region surrounding the roots, is a dynamic environment where plants and microorganisms engage in continuous biochemical exchanges. Root exudates attract and sustain a wide range of microbes that, in return, participate in nutrient cycling, pathogen suppression, and stress mitigation. This microenvironment is enriched with diverse bacterial genera such as *Agrobacterium*, *Azospirillum*, *Azotobacter*, *Burkholderia*, *Bacillus*, *Chromobacterium*, *Caulobacter*, and *Erwinia*, all of which are recognized for their plant-growth-promoting attributes (Verma et al., 2019).

Based on their ecological roles and interactions with orchid hosts, orchid-associated microbes can be broadly categorized into four groups:

- Orchid Mycorrhizal Fungi (OMF): The orchid-associated fungus forms coiled structures called pelotons within orchid cells’ hyphae, facilitating nutrient exchange in the symbiotic relationship.
- Root-Associated Bacteria (RAB): Bacteria present in the rhizosphere or near the root of orchids. These bacteria significantly influence plant health and growth, interacting with both the plant and beneficial microbes like mycorrhizal fungi to support overall ecosystem balance (Kaur and Sharma, 2021).
- Mycorrhizal Helping Bacteria (MHB): The class of bacteria that facilitate the growth and development of mycorrhizal association by enhancing spore germination, hypha, and root branching (Yang et al., 2023).
- Plant Growth Promoting Bacteria (PGPB), colonize plant tissues harmlessly and support plant health by enhancing nutrient uptake, disease resistance, stress tolerance, and growth through mechanisms like nitrogen fixation and hormone production. Their use in agriculture offers a sustainable way to boost crop productivity, reduce chemical fertilizer dependence, and promote environmental resilience, highlighting the potential of microbial communities for eco-friendly plant growth (Gupta et al., 2024; Mazoyon et al., 2023; Özbudak and Bilgin, 2024; Su et al., 2024).

This review aims to examine the intricate and dynamic relationships between orchids and plant growth-promoting bacteria (PGPB). It highlights the diverse roles these bacteria play in supporting orchid germination, growth, nutrient acquisition, and resilience to environmental stressors. The main objectives of this review are to:

- Summarize the diversity of bacterial communities associated with orchids, including both common and less explored strains, and highlight their contributions across different developmental stages.
- Describe the functional traits and mechanisms through which PGPB influence orchid physiology, such as nutrient mobilization, hormone regulation, stress tolerance, and protection against pathogens.

Highlight the protective roles of orchid-associated bacteria, particularly their antagonistic effects on phytopathogens and their contributions to plant defence.

- Provide an integrative overview of current knowledge and recent findings on orchid–bacteria interactions, emphasizing their ecological importance and potential applications in orchid conservation, cultivation, and sustainable horticulture.

## 2 Methods for identifying and studying plant growth promoting bacteria (PGPB)

The interaction of orchids with their associated microbial communities is diverse and complex, involving organisms from multiple kingdoms. Although mycorrhizal fungi have long been recognized as essential partners in orchid germination and early development, this represents only one component of the orchid microbiome. In contrast, the associations between orchids and bacteria—along with the mechanisms, functional traits, and adaptive roles of these bacterial partners—remain far less explored. Understanding these bacterial interactions is critical, as emerging evidence shows that plant-associated bacteria significantly influence orchid nutrition, growth, stress tolerance, and overall ecological fitness (Thiergart et al., 2020). However, recent advancements in molecular and adaptive culture-based techniques have facilitated not only the exploration of bacterial diversity but also shed light on the intricate interactions with the ecosystem. Bacterial endophytes exhibit a remarkable diversity not only in the plant ecosystem but also in the plant tissue. Unlike the controlled environment of laboratory cultivation, where conditions are manipulated for specific experiments, the natural interactions between plants and endophytes foster a broad array of microbial species. This diversity stems from the dynamic interplay of plant genetics, environmental factors, developmental stage, and the unique niches within the host (Goulart et al., 2019). In contrast, lab cultivation, with its controlled parameters, tends to limit microbial diversity. Bacterial endophytes exhibit a more diverse range of species when studied through culture-independent methods compared to culture-dependent approaches. The utilization of culture-dependent studies has paved the way for advanced molecular analyses, including next-generation sequencing and metagenomic analysis (Adeleke et al., 2023; Balogun et al., 2023; Sohaib et al., 2024; Verstraete et al., 2023; Yuan et al., 2023). These techniques significantly enhance our comprehension of functional diversity, composition, taxonomic, and genetic variations within bacterial endophyte communities (Staley and Sadowsky, 2018). By embracing new advanced approaches, researchers can uncover a broader spectrum of microbial diversity, contributing to a more comprehensive understanding of the intricate ecosystems formed by bacterial endophytes. This shift in methodology allows for a more nuanced exploration of their ecological roles and potential applications in various fields (Lema et al., 2023).

**Figure 1:**
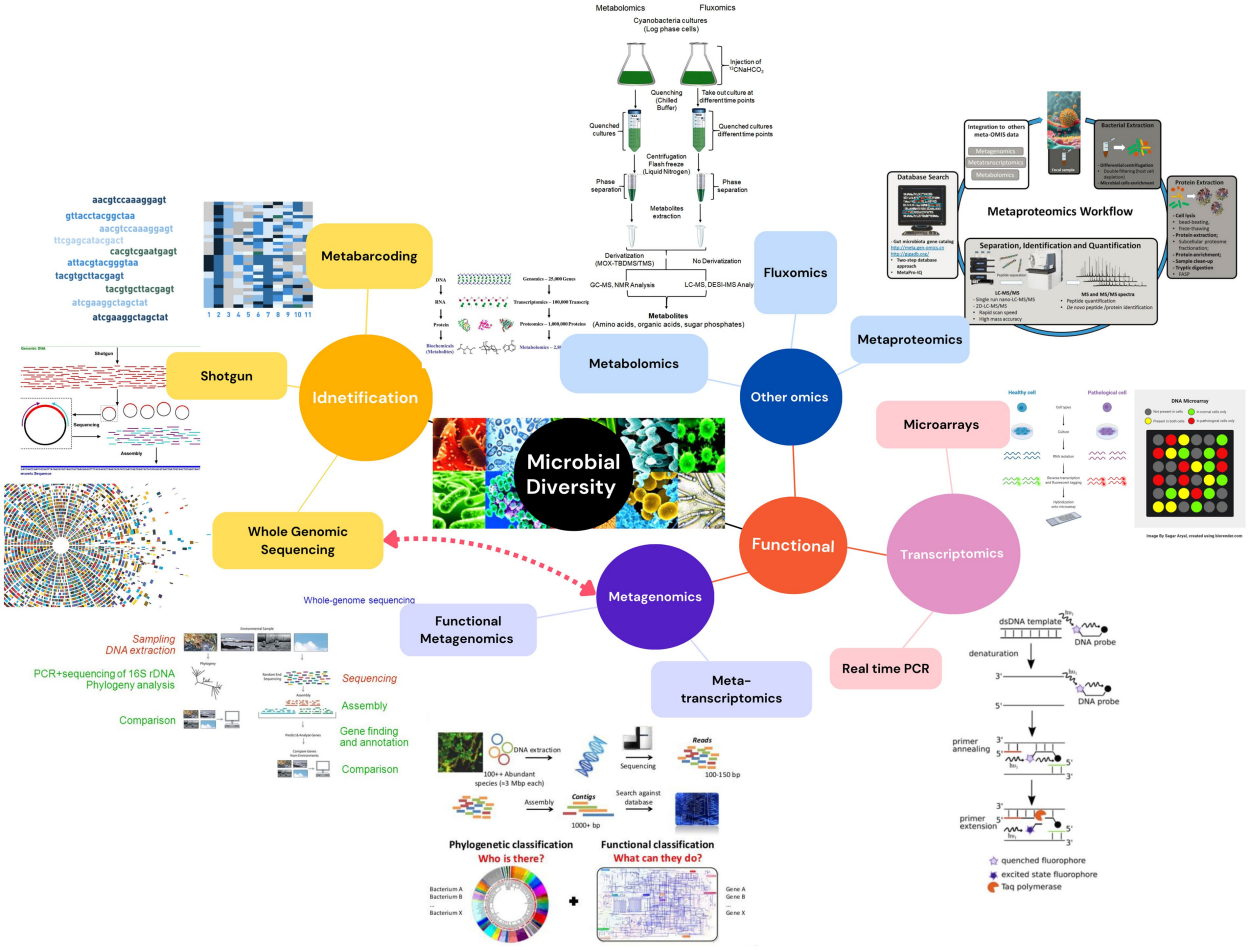
Approaches used in the identification and functional study of PGPB diversity.

The identification of bacterial isolates primarily depends on genotype-based techniques, which are broadly classified into two categories. Sequence-based methods such as genome shotgun sequencing, metabarcoding, and proteomics offer detailed genetic information, while pattern- or fingerprint-based techniques including phospholipid fatty acid analysis, denaturing gradient gel electrophoresis (DGGE), single-strand conformation polymorphism (T-RFLP), and amplified fragment length polymorphism (AFLP) analyze genetic patterns. As illustrated in Figure 1, these approaches enable bacterial identification by comparing the degree of similarity among organisms, supporting the development of robust reference databases as shown in Table 1. Molecular identification of bacteria involves a variety of genes (Lau et al., 2013) such as 16S-23S rRNA intergenic space (ITS), rRNA 23S (Karmakar et al., 2023), *Rpo*B (*β* subunit of RNA polymerase) and *Gyr*B (β subunit of DNA gyrase) (Basavand et al., 2022; Kumar et al., 2023).

**Table 1:** Diversity of bacteria associated with orchid plants, habitat, and identification method.

Sr. no	Orchid plant	Location	Host organ	Identification method	Isolated taxa	References
1	*Vanilla planifolia*	Mexico	Roots and stems	16S and ITS rDNA metabarcoding	Proteobacteria, Actinobacteria, Bacteroidetes, Acidobacteria, and Planctomycetes	Carbajal-Valenzuela et al. (2022)
2	*Chloraea barbata, Chloraea collicensis, Chloraea gavilu, Chloraea magellanica, Gavilea Araucana, Gavilea lutea*	Southern Chile	Roots (pelotons)	16S rDNA gene and PCR	Proteobacteria (Pseudomonadales, Burkholderiales, and Xanthomonadales)	Herrera et al. (2020a)
3	*Phalaenopsis* sp.	Taiwan	Plant	PCR testing using Dickeya-specific primers	*Dickeya spp*	Wei et al. (2021)
4	*Microcoelia moreauae, Chiloschista parishii*	Green house conditions	Roots	e 16S rRNA, DGGE analysis	*Cyanobacteria; Oscillatoriales* (*Microcoleus* sp., *Oscillatoria* sp., *Pseudophormidium* sp.), *Synechococcales* (*Leptolyngbya* sp., *Pantanalinema* sp.)*, Nostocales* (*Komarekiella* sp.)	Tsavkelova et al. (2022)
5	*Dendrobium moniliforme*	–	Roots	16S rRNA gene and PCR	Acidobacteriota and Verrucomicrobiota, Actinobacteriota, Bacteroidota, Proteobacteria *(Alphaproteobacteria and Gammaproteobacteria), and Firmicutes*	Nishioka and Tamaki (2022)
6	*Dendrobium* sp.	–	Roots	16S rRNA and MiSeq sequencing	*Flavobacterium* sp. *strain GSB-24*	Nishioka et al. (2023)
7	*Dendrobium* sp.	–	Roots	16S rRNA and MiSeq sequencing	*Dyella* sp. *strain GSA-30.*	Nishioka et al. (2023)
8	*Dendrobium christyanum, D. formosum, D. kentrophyllum, D. findlayanum, D. chrysanthum, Calanthe cardioglossa, D. friedericksianum, D. chrysotoxum, D. crumenatum, D. heterocarpum, Coelogyne lawrenceana, Eria ornate, Cleisostoma rostratum, Coelogyne assamica, and Pinalia globulifera*	Thailand	Roots	16S rRNA and PCR	*Streptomyces* (*S. parvulus, S. tendae, S. ardesiacus, S. heilongjiangensis, and S. daghestanicus, S. antibioticus, S. malaysiensis, S. deserti, S. spiralis, S. thermoviolaceus subsp. apingens, S. globosus, S. collinus, S. olivaceus, and S. zaomyceticus). Micromonospora (M. humi, M. maritima, M. tulbaghiae, M. schwarzwaldensis, M. chersina, M. chalcea M. citrea and M. aurantiaca). Streptosporangium (S. sandarakinum and S. pseudovulgare) Actinomadura hibisca. Streptomyces, Micromonospora and Streptosporangium*	Tedsree et al. (2022)
9	*Spathoglottis plicata, Dendrobium sagittatum, Malaxis kobi, Apendicula alba, Pholidota carnea, Dendrobium tenellum, and Bulbophyllum compressa*	Indonesia	Roots	16S rRNA in V3 and V4 (for bacteria), PCR specific primers 341 and 806R	*Proteobacteria*, Firmicutes, Bacteriodota, and Actinobacteriota*. Bacterial genera: Pseudomonas, Serratia, Rhodanobacter, Acinetobacter, Escherichia, Bifidobacterium, Clostridium, Parabulkholderia, Faecalibacterium, and Muribaculaceae*	Pangastuti et al. (2023)
10	*Gastrodia elata*	Shaanxi province, China	Soil	16S rRNA and PCR specific primers 515F and 806R	*Pseudomonas, Novosphingobium*, *Flavobacterium, Rahnella, Variovorax, Bradyrhizobium, Massilia, Collimonas, Duganella, Raoultella, Limnohabitans, Dyella, Rhizobacter and Mucilaginibacter*	Liu et al. (2022)
11	*Doritaenopsis*	–	Flower stalk	Whole genomic sequencing by PacBio RS long-read sequencing platform and Illumina Hiseq 2000 platform	*Mycobacterium Mya-zh01*	Pan et al. (2020)
12	*Epipactis albensis, E. helleborine and E. purpurata*		Closed flower buds, stem, rhizome fragments and adventitious roots.	Proteomics-based MALDI	*Bacillus* spp., *Clostridium* spp., *Pseudomonas* spp. and *Stenotrophomonas* spp	Jakubska-Busse et al. (2021)
13	*Platanthera cooperi and Platanthera praeclara*	California	Root and soil	Sequencing of V4 and V5 regions of the 16S rRNA gene.	*Pseudomonadaceae, Burkhol-deriaceae, Thermaceae, and Rhodanobacteraceae*	Kaur et al. (2023)

Next, Generation Sequencing (NGS), or high-throughput sequencing, encompasses advanced technologies that allow rapid, simultaneous sequencing of millions of DNA or RNA fragments. Unlike traditional Sanger sequencing, NGS offers greater speed, scalability, and efficiency, resulting in significantly higher data output and broader applications in genomic research and analysis. The categorization of NGS technologies into short-read (second-generation) and long-read (third generation) paradigms, based on read length, is a key focus. These technologies, with high accuracy, cost-effectiveness, and high-throughput capabilities, have been widely used in various applications, although their limitation lies in the relatively short read lengths (Hu et al., 2021). Advances in base-calling algorithms, sequencing chemistry, and error correction methods are enhancing data accuracy and reliability, thereby increasing output, improving efficiency, and expanding applications in genomics research (Kchouk et al., 2017).

Metagenomics provides a powerful lens for PGPB-orchid research by capturing the full spectrum of microbial diversity including bacteria, fungi, and archaea, allowing precise identification and functional analysis of microbial communities. This approach unveils intricate orchid–microbe interactions, enhances ecological understanding, and supports conservation efforts. Following sequencing, data analysis relies on a range of dynamic and continuously updated databases essential for genomic annotation, gene mining, and functional interpretation. These resources help researchers uncover gene functions, explore biological pathways, and keep pace with emerging insights, as illustrated in Table 2 and Figure 1.

**Table 2:** Different orchid and bacterial metagenomic databases.

Data base name	Content	Website	References
OrchidBase 5.0	Orchidaceae Plant Genome Molecular Database	https://cosbi.ee.ncku.edu.tw/orchidbase5/	Chen et al. (2022)
Main lab for bioinformatics	Analytical, computational tools and database for crop genomics, sequence analysis pipelines and genomic regions and marker controlling traits	https://www.bioinfo.wsu.edu/	Cao (2023), Xu et al. (2023)
Genome Sequence Annotation Server (GenSAS)	GenSAS, online platform for whole genome, structural annotation, functional annotation of eukaryotes and prokaryotes. Genome sequences, repeat masking, gene model prediction, structural features, it integrates JBrowse and Apollo integration for visualization and editing.	https://www.gensas.org/	Humann et al. (2019)
Planteome	Gene profiling, phenotypes in OMICs and developing annotation standards with other genomic databases for plant biology	https://planteome.org/	Cooper et al. (2023)
GENEVESTIGATOR	Transcriptome data mining and comparison platform	https://genevestigator.com/	Tahmasebi et al. (2021)
WoLFPSOT	Computational tools for protein sequencing, structuring, and finding domains for bacteria, fungi, insects, and mammals.	https://www.genscript.com/wolf-psort.html	Zheng et al. (2019)
EasyGo	Provide functional annotations for many genes as well as probe sets from expression microarrays.	http://bioinformatics.cau.edu.cn/easygo/	Fahmideh et al. (2023), Zhao et al. (2023)
GOMAP	Annotate plant protein sequences and compile the annotations together to generate an aggregated dataset.	https://bioinformapping.com/gomap/	Wimalanathan et al. (2021)
NGS and sRNA	Next generation sequencing (NGS) database including small RNA sequencing, sequencing of cleaved target RNAs	https://mpss.meyerslab.org/	Eckardt et al. (2023a), Eckardt et al. (2023b), Li et al. (2023)
Microbial genome database (MBGD)	Provide gene function with their expression and species evolution that reveals their pathogenic molecular mechanism and elucidate the evolutionary relationship between bacteria and the internal structure of the genome.	http://mbgd.genome.ad.jp/	Uchiyama et al. (2019)
Global Catalogue of Metagenomics (gcMeta)	Updated monthly, provides 90 tools which can preprocessing, assembly, structure, annotation, metagenome analysis, comparative analysis, and visualization of microbial meta-omics.	https://gcmeta.wdcm.org/	Shi et al. (2019)
High quality Ribosomal RNA Databases (SILVA)	Provides analysis service for rRNA gene (rDNA) amplicon reads obtained from high-through put sequencing (NGS) approaches based on an automatic software pipeline to combine alignment, search and classify as well as reconstruction of trees in a single web application.	https://www.arb-silva.de/	Glöckner (2019)
National Center of Biotechnology information (NCBI)	DNA sequences, Gen bank, data for computational biology and bibliographic data	https://www.ncbi.nlm.nih.gov/	Renner et al. (2023), Wang et al. (2023)
MIMt	Determination and quantification of the taxonomic composition of microbial communities.	https://mimt.bu.biopolis.pt/	Cabezas et al. (2023)

## 3 Factors shaping orchid-associated bacterial diversity

### 3.1 Orchid species and organ-specific bacteria

The microbiota shows distinct phylogenetic patterns across different plant species and organs in three orchid species, *Neottia ovata*, *Spiranthes spiralis*, and *Serapias vomeracea*, focusing on leaves, stems, capsules, and roots. Proteobacteria and Actinobacteria were the dominant bacterial groups, with *Cutibacterium* as the most representative genus. Other common endophytic genera included *Acinetobacter*, *Pseudomonas*, and *Rhizobium*, while *Thermus* was an unusual finding in plant microbiota. Analysis of microbial diversity showed that plant organ type, rather than species, primarily shaped bacterial communities. Notably, *N. ovata* and *S. vomeracea* shared similar microbiota despite different habitats. Microbial richness and diversity decreased from roots to capsules, suggesting that orchids actively select specific bacterial taxa, increasing specialization in above-ground parts. These findings challenge previous assumptions about species-based microbial clustering and emphasize the need to consider individual plant organs in microbiome research (Alibrandi et al., 2020).

Extensive study was conducted to examine the bacterial endosymbiotic 16/18S rRNAs from the stem and roots of *Dendrobium officinale* by PCR-DGGE. The crucial aim of the research was to design a primer pair to identify the bacteria and their roles within the *D. officinale*. To analyze endophytic bacteria in *Dendrobium officinale*, a novel primer pair, fM1/rC5 was designated to specifically target the endophytic bacterial 16S rRNAs while excluding chloroplast and mitochondrion 16S/18S rRNAs of the plant. The primer pair demonstrated perfect specificity, distinguishing endophytic 16S rRNAs from plant organelle rRNAs, and exhibited broad universality across different bacterial genera and species from 19 phyla. Among the identified genus, the more commonly dominant genera were *Burkholderia*, *Sphingomonas* and Pseudomonas. The findings revealed varying diversities in both roots and stems of the plants across all three locations (Yu et al., 2013).

The dominant shared bacteria included *Acidobacteria, Actinobacteria, Bacillota, Bacteroidetes* and *Proteobacteria* were identified from the roots, stems and leaves of medicinal orchid, *Dendrobium nobile*. Notably, roots exhibited a more abundant and diverse microbiome, with potentially beneficial bacteria for plant growth. This diversity in roots, crucial for nutrient acquisition, suggests a strategic recruitment of microbes involved in nutrient metabolism and phytohormone synthesis, enhancing the plant’s ability to thrive in challenging environments. This establishes an endophytic microbial interaction network based on bacterial abundance, highlighting the coexistence of numerous endophytes within the same phylum. Notably, a favorable relationship is observed among beneficial taxa, indicating a potential role in recruiting beneficial genera and enriching the functional diversity of the host (Zhao et al., 2023). Although this study unveils the structural and potential functional characteristics of endophytic bacteria, the precise mechanism of intracellular endophyte internalization remains unknown and further investigation is necessary to understand the intricate interactions in orchids.

The study of *D. officinale* microbiota revealed that culture-dependent methods detected Firmicutes, Proteobacteria, and Actinobacteria, while DGGE primarily identified Firmicutes and Proteobacteria, with most genera belonging to Proteobacteria (e.g., *Brevundimonas*, *Burkholderia*, *Klebsiella, Rhizobium*). Metagenomic analysis confirmed Proteobacteria as the dominant phylum across all tissues, along with varying levels of Firmicutes, Actinobacteria, and Bacteroidetes. The most abundant genera included *Aquamicrobium*, *Brucella*, *Pseudochrobactrum*, *Burkholderia*, and *Acinetobacter*. These findings underscore the consistent dominance of Proteobacteria and highlight the differences in microbial composition among tissues. The study also points out the limitations and biases of culture-dependent methods, advocating for a combined approach using both culture-based and molecular techniques for a more complete understanding of microbial diversity in *D. officinale* (Pei et al., 2017).

**Figure 2:**
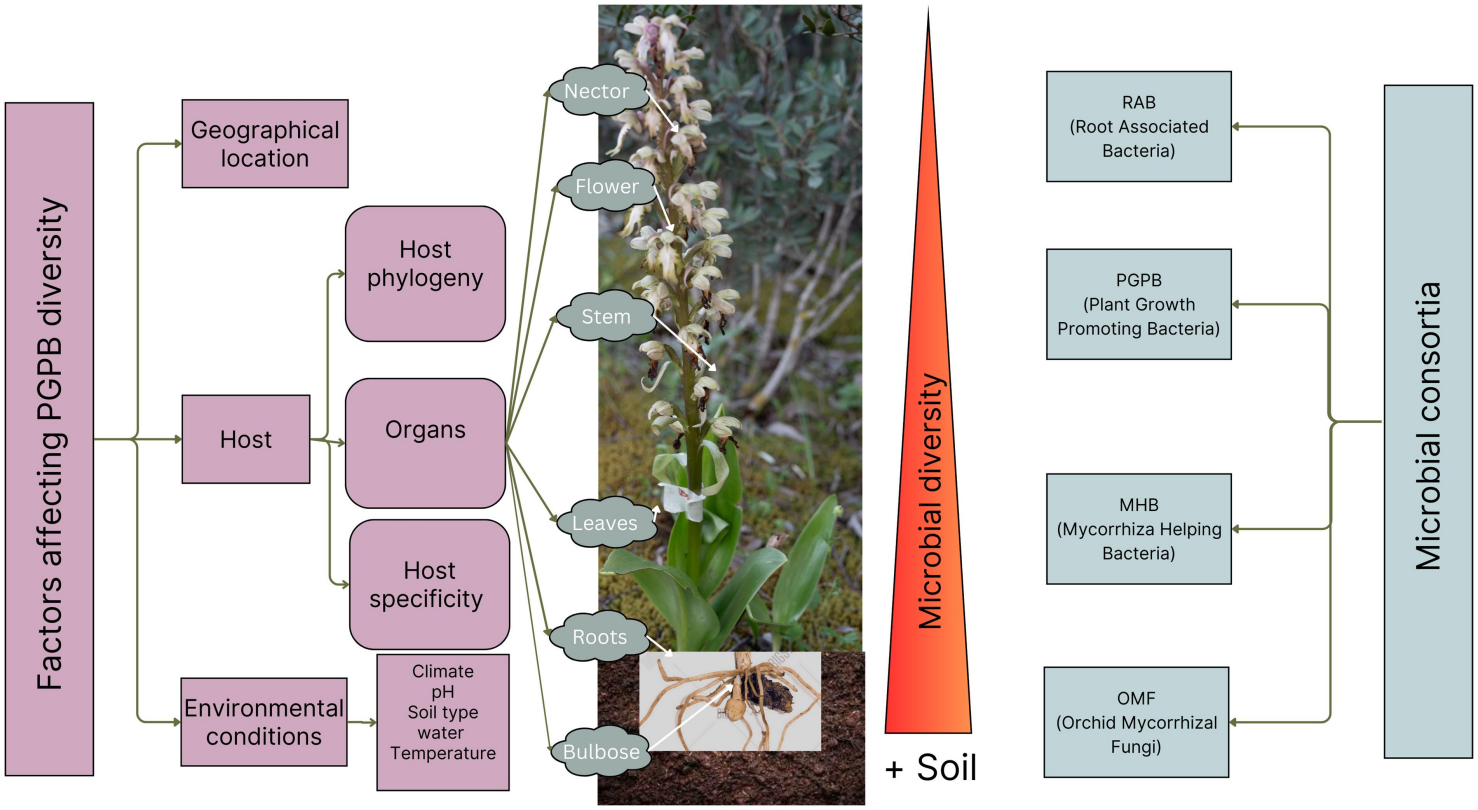
Diversity gradient of microbes and the factors affecting the distribution and composition of PGPB.

The current knowledge about microorganisms in orchid floral nectar is limited. Diverse bacterial communities were identified in the nectar of seven *Epipactis* species, *E. atrorubens, E. helleborine, E. purpurata, E. microphylla, E. muelleri, E. neglecta,* and *E. palustris* using PCR and sequencing. The dominant bacterial phyla included *Actinobacteria (Dermococcaceae, Microbacteriaceae), Bacteroidetes (Chitinophagaceae), Proteobacteria (Enterobacteriaceae, Methylobacteriaceae, Moraxellaceae, Pseudomonadaceae, Sphingomonadaceae), Firmicutes (Bacillaceae, Leuconostocaceae, Paenibacillaceae, Staphylococcaceae),* with limited phylogenetic diversity but with consistent pattern of microbial community structure across environments. These microbes may influence pollination by altering nectar chemistry and pollinator behavior. However, the presence of compounds like oxycodone in nectar implies other factors also affect pollinator interactions. The exact role of microbes in orchid reproductive success remains unclear, and future research should explore the complex relationships between orchids, nectar-inhabiting microbes, and pollinators for a more holistic approach (Jacquemyn et al., 2013). Figure 2 illustrates the microbial diversity gradient across plant organs, highlighting how environmental and anatomical factors influence the distribution and composition of PGPB. These patterns align with observed microbial specialization and functional adaptation in orchid-associated communities.

### 3.2 Influence of geography on orchid-associated bacteria

The bacterial and diazotrophic diversity in *Dendrobium catenatum* was studied by Li et al. (2017) using 16S rRNA and meta-nifH gene sequencing, where high-throughput sequencing via Illumina GAIIx identified 699 bacterial genera across 22 phyla and 45 diazotrophic genera from 4 phyla. The study investigated microbial community relationships through an unweighted heatmap. Community analysis revealed that stem and leaf samples clustered together, while roots formed a separate group, indicating distinct microbial compositions between above- and below-ground tissues. Proteobacteria were the dominant phylum (71.81–96.08%), with key genera including *Delftia*, *Pseudomonas*, *Burkholderia*, *Escherichia/Shigella*, and *Sphingomonas*. These bacteria, particularly *Burkholderia*, *Pseudomonas*, and *Sphingomonas*, are commonly found in *Dendrobium* and other orchids and are known to support plant health (Li et al., 2017). The higher abundance of *Delftia* and *Escherichia/Shigella* needs further investigation due to their opportunistic pathogenic nature. Environment- and tissue-specific bacteria highlight the role of substrate and plant tissue in shaping microbial communities, underscoring environmental influence on bacterial diversity and the need for further validation.

In a separate study was performed, root endophytic bacteria were isolated from *Dendrobium officinale* growing on tree trunks (*Albizia julibrissin*, *Cyclobalanopsis myrsinifolia*) and sedimentary rocks. Samples were grouped by substrate (Groups 1–3 for trunks, Group 4 for rocks) and analyzed using high-throughput sequencing. While alpha diversity showed no significant differences in overall richness and evenness among groups, beta diversity analysis (PCA) revealed variations in community structures. Specific genera were associated with each group, such as *Spirosoma* and *Staphylococcus* (Group 1), *Mucilaginibacter* and *Clostridium sensu stricto* (Group 2), *Terriglobus* (Group 3), and *Pseudomonas*, *Bacillus*, and *Delftia* (Group 4). These findings suggest both commonality and variability in the composition of endophytes in the roots of arboreal and lithophytic *D. officinale*, reflecting the influence of growth substrate on microbial composition (Li et al., 2023). The investigation into the endogenous relationship between bacterial diversity and habitat provides insights into the adaptation and evolution mechanisms of orchid plants.

In the case of *Phalaenopsis pulcherrima*, an endemic Southeast Asian orchid species, 16S rDNA sequence analysis revealed the isolated endophytic bacteria and classified them into seven genera, including *Agrobacterium, Bacillus, Burkholderia, Erwinia, Paenibacillus, Pandoraea* and *Pantoea*. *Bacillus* emerged as the dominant genus, followed by *Pantoea* and *Burkholderia*. The study revealed significant differences in the endophytic bacterial community structures of *P. pulcherrima* in distinct habitats, with *Bacillus* and *Pantoea* dominating in land-borne populations and *Bacillus* and *Burkholderia* in stone-borne populations (Fang-Fang et al., 2016). The same results are supported by other findings which showed that the microbiome variation in *G. conopsea* is influenced by geographical location, developmental stage, and compartment. Predominant bacterial taxa identified include Proteobacteria, Bacteroidetes, Acidobacteria, Actinobacteria, Verrucomicrobia, Chloroflexi and Planctomycetes (Lin et al., 2020). Consideration of compartment and developmental stage is crucial for analyzing microbiota composition variation. Despite significant microbial composition differences across locations, *G. conopsea*’s symbiotic microorganisms exhibit a degree of specificity. Table 3 complements the text by summarizing the bacterial diversity linked to orchid species, their habitats, and detection techniques. It highlights key microbial taxa and identification methods, reinforcing the role of habitat in shaping microbial communities.

**Table 3:** Orchid related PGPB and their role.

Sr. No.	Isolated bacteria	Orchid plant	Selected biological function	References
1	*Bacillus* spp	*Vanda cristata* (leaves)	IAA, antimicrobial compounds production	Shah et al. (2021)
2	*Streptomyces, Actinomadura*, *Nocardia*, *Nocardiopsis*, *Nocardioides, Pseudonocardia*, *Microbacterium*, and *Mycolicibacterium*.	*Dendrobium nobile*, *Dendrobium chrysotoxum*, *Dendrobium moschatum*, *Dendrobium densiflorum*, *Dendrobium fugax*, *Rhynochostylis retusa*, *Vanda corulea*, *Renanthera imschootiana*, *Micropera obtusa*, *Cymbidium eburneum*.	IAA production, phosphate solubilization, ammonia production, siderophore production, ACC deaminase activity, extracellular enzyme (proteases, chitinase, Cellulase, pectinase), antifungal activity	Saikia et al. (2022)
3	*Pseudomonas fluorescens*	*Bletilla striata (roots)*	Nitrogen fixation ability, IAA production, phosphate solubilization, siderophore production	Wu et al. (2022)
4	*B. halotolerans, Bacillus* sp.*, Entrobactor* sp.	*Dendrobium* sp.	IAA production Phosphate solubilization ACC deaminase activity, siderophore, ammonia production,	Sarawaneeyaruk (2023)
5	Garm negative bacteria	*Vanda*	IAA production	Inkaewpuangkham et al. (2022a)
6	*Genus Pantoea, Brevibacterium, Achromobacter, Arthrobacter, Klebsiella, Mixta, Bacillus, and Pseudomonas.*	*Rhynchostylis retusa*	Phosphate solubilization, IAA production, siderophore, HCN biofilm and ammonia production.	Yadav et al. (2023)
7	*Mycobacterium strain Mya-zh01*	*Doritaenopsis*	IAA production	Pan et al. (2020)
8	*Bacillus toyonensis, Bacillus mobilis, Pseudomonas fluorescens, Acinetobacter calcoaceticus, Bacillus simplex, Pseudomonas frederiksbergensis, Bacillus thuringiensis, Pseudomonas baetica, Agrobacterium tumefaciens, Bacillus cereus, Stenotrophomonas maltophilia, Bacillus mobilis, Bacillus cereus, Paenibacillus* sp.	*Limodorum abortivum, Spiranthes spiralis, Orchis coriophora, Ophrys sphegodes, Anacamptis pyramidalis, Orchis coriophora, Spiranthes spiralis, Orchis tridentata, Serapias vomeracea, Himantoglossum caprinum, Platanthera bifolia*	IAA production, Phosphate solubilization ACC deaminase activity	Altinkaynak and Ozkoc (2020)
9	Endophytic bacteria	*Vanda*	IAA production and nitrogen fixation	Inkaewpuangkham et al. (2022b)
10	*Bacillus* spp.	*Vanda cristata*	IAA production	Shah et al. (2021)
11	*Bacillus subtilis*	*Vanda cristata*	Auxin production	Chand et al. (2023)
12	*Bacillus velezensis*	*Habenaria lindleyana, Habenaria rhodocheila and Pecteilis sagarikii*	IAA production, antagonistic bacteria against *Acidovorax avenae*	Akarapisan et al. (2020)
13	*Streptomuces aureus, S. antimycoticus, S. aureocirculatus*	*Serapias* sp.	Antimicrobial activity and biofilm inhibition test	Ateş (2023)

### 3.3 Environmental factors shaping orchid-associated bacterial communities

The identified 478 PGPB with 195 strains associated with leaves (LA), 283 strains in rhizospheric soil (SA), and 95 strains from non-plant environments (OA) were investigated. The study identified eight genera, with *Bacillus, Burkholderia, Sphingomonas, Methylobacterium, Pseudomonas, Klebsiella, Rhizobium* and *Serratia*. Using hierarchical clustering based on CAZyme and secondary metabolite genes, strains were grouped by habitat, revealing a strong association between gene profiles and environmental origin. Phylo-PCA of 163 core genes demonstrated a correlation between gene abundance and habitat characteristics, indicating their role in environmental adaptation (Wang et al., 2023).

A significant knowledge gap regarding the rhizobium and endophytic communities of *Dendrobium officinale*, an epiphytic orchid threatened by extinction and harsh growth conditions were found. Using metagenomic sequencing of samples from Mount Danxia, it was found that the rhizosphere selectively enriched specific bacterial groups, namely Proteobacteria, Acidobacteria, Actinobacteria, Bacteroidetes, and Cyanobacteria with Proteobacteria and Acidobacteria being predominant. The clear distinction between the microbial communities of the rhizosphere and endosphere highlighted unique microbial preferences, aligning with findings from other studies on common endophytes (Zuo et al., 2021).

Notable plant growth-promoting genera were identified in both the rhizosphere and endosphere, indicating distinct microbial roles across compartments. PCA and NMDS analyses showed clear separation between rhizosphere and endosphere communities, suggesting compartment-specific microbial preferences, with the endosphere harboring more shared endophytes. Dominant rhizosphere genera included *Bradyrhizobium*, *Enterobacter*, *Frankia*, *Klebsiella*, *Leclercia*, *Massilia*, *Paenibacillus*, *Pseudomonas*, and *Streptomyces*. The root endosphere was enriched with *Tulasnella* and *Serendipita*, while the stem endosphere featured *Colletotrichum* and *Burkholderia*. Other key endosphere genera included *Paraburkholderia*, *Rhizophagus*, and *Acetobacter*. Functional profiling using KEGG, eggNOG, and CAZy databases revealed enriched metabolic and signalling pathways, highlighting microbial contributions to plant adaptation and stress tolerance (Wang et al., 2022).

The study of roots and soils of orchid species, *Epipactis atrorubens*, *Platanthera bifolia*, *P. longifolia*, and *E. pontica from* extreme habitats like mining dumps, highlighting their adaptation to harsh, nutrient-poor, humus-lacking conditions. Illumina MiSeq sequencing identified 30 bacterial species across eight phyla, with *Candidatus Udaeobacter* dominant in Pyrinomonadaceae in Dobšiná. Key microbes included *Trichophaea pseudogregaria, Gemmata,* and acid-tolerant taxa. The findings suggest that microbial presence, especially mycorrhizal fungi, rather than species specificity, supports orchid survival in harsh, anthropogenic environments, emphasizing the Orchidaceae family’s remarkable ecological adaptability (Böhmer et al., 2020).

The relationship between fungi and bacterial strains isolated from the roots of *Dendrobium catenatum in vitro* cultural conditions was investigated. In this study, while the potential presence of endophytic bacteria associated with the fungal inoculum is acknowledged, the observed promotion of plant growth is attributed to direct effects on seedling biomass and the development of above and underground plant parts by fungal and bacterial strains, respectively. The findings suggest distinct roles of the co-inoculation of these two microorganisms can positively influence the different aspects of plant growth (Wang et al., 2016).

The diversity of bacteria associated with orchid species varies widely depending on their habitat, plant part, and identification techniques used. To provide a clearer understanding of this microbial diversity, Table 1 summarizes key bacterial genera reported from different orchid species, their respective habitats (terrestrial, epiphytic, or lithophytic), and the molecular or culture-based methods employed for their identification. This compilation supports the discussion on the ecological roles and taxonomic range of orchid-associated bacteria presented in the text.

## 4 Mechanisms of host–microbe interaction

Orchids exhibit mycoheterotrophy, forming common associations with a diverse array of fungi and various free-living or endophytic microorganisms. These organisms manifest different life cycles, including saprophytic and phytopathogenic ones, often involving *Fusarium, Thanatheporus,* and many septate endophytes (Hartvig et al., 2024; Venice et al., 2024; Wang et al., 2024). Additionally, it is crucial to investigate and demonstrate various plant growth-promoting traits exhibited by plant related bacteria. These traits can offer valuable insights into the mechanisms behind inhibitory effects on the growth of plant pathogenic fungi. Nevertheless, further research is required to comprehend the specific role of root-associated bacteria in the physiology of fully mycoheterotrophic plants (Herrera et al., 2020a).

While the concept of plant-bacteria association has been recognized for some time, a thorough understanding of the mechanisms employed PGPB poses challenges. The intricacies of these mechanisms make it elusive to harness them effectively for consistently enhancing plant growth in natural environments. Despite ongoing research efforts, the complexities involved in deciphering and leveraging these intricate interactions underscore the challenges in translating scientific knowledge into practical applications for sustainable plant growth. Achieving a more nuanced understanding of the nuanced interplay between plants and growth-promoting bacteria is essential for unlocking their full potential in natural ecosystems.

Orchid associated bacteria have many direct and indirect effects on the germination, development, and growth of the orchid plant. This emphasizes further study and investigation of functional diversity of plant growth promoting bacteria to understand their functional role and mechanisms in the plant life cycle and agroecosystem. The indirect effect includes production of phytohormones, i.e., IAA, ABA, Salicylic acid and include nutrient uptake etc., while indirect effects include biocontrol and control of toxins etc. Understanding the mechanisms of bacteria-orchid symbiosis is crucial to fully comprehend the impacts of endophytic bacteria on orchid reproduction. This understanding will aid in developing new strategies for orchid protection and better utilization of their medicinal principles.

### 4.1 Production of biomolecules to enhance germination and development

The phytohormones can be categorized into two types based on their functions: growth and regulatory hormones (such as salicylic acid (SA), indole acetic acid (IAA), and Zeatin) and stress resistance hormones [including 1-aminocyclopropane-1-carboxylate (ACC) deaminase and Abscisic Acid (ABA)].

#### 4.1.1 Modulating plant indole acetic acid levels

IAA is the most active phytohormone that plays vital role in many physiological processes like seed germination, organogenesis, tropism responses, and gene regulation mostly via signaling mechanism (Ryu and Patten, 2008). IAA can also initiate lateral and adventitious root formation and mediate many other hormones like ethylene to cope with stress conditions. In the realm of orchids, auxins play a vital role in various physiological processes essential for plant development. These include promoting root and shoot growth, facilitating the formation of protocorm-like bodies (PLBs), and contributing to the successful germination of orchid seeds (Fang et al., 2022). The presence and regulation of auxins are crucial factors influencing the intricate life cycle and growth patterns of orchids.

Specific trends were observed in *Dendrobium moschatum*, where both common PGPR (e.g., *Azospirillum*, *Enterobacter*, *Streptomyces*) and lesser-known genera (*Roseomonas*, *Agrococcus*) were evaluated for auxin production. In *Dendrobium nobile*, bacterization using various endophytes (e.g., *Mycobacterium* sp., *Bacillus pumilus*) revealed orchids’ broad microbial associations, though with limited preference for *Streptomyces* and *Azospirillum*. In contrast, *Agrococcus* and *Sphingomonas* significantly promoted seed germination (Tsavkelova et al., 2016).

The lower amount of IAA can enhance plant root growth, but the production in higher quantities can be responsible for stunt growth (Parperides et al., 2021). The enhancement of host plant growth by endophytic bacteria is not solely reliant on IAA production. Conversely, the reverse process, the degradation of IAA, can also exert a notable influence on promoting plant growth. The *R. retusa* aerial root associated bacteria were identified *as Microbacterium testaceum* based on 16S rRNA analysis. The identified bacterial strain produces an efficient amount of IAA that enhances the orchid growth and development by various growth-promoting properties (Yadav et al., 2022).

Numerous studies have highlighted the auxin-producing capabilities of bacterial species associated with orchids, including *Bacillus, Enterobacter, Pseudomonas, Stenotrphomonas,* and *Microbacterium.* Notably, the most prolific auxin producers, such as *Pseudomonas, Serratia, Stenotrophomonas, Rhizobium,* and *Enterobacter*, primarily belong to the gram-negative class (Panigrahi et al., 2020). However, certain gram-positive bacteria, including *Microbacterium, Bacillus*, and *Streptomyces*, also exhibit high productivity in IAA production (Myo et al., 2019).

Practically speaking, treating orchid seeds with strains that produce IAA could be a beneficial and advantageous approach for *in vitro* orchid propagation. Several findings directly affirm that IAA originating from microbes significantly contributes to promoting orchid germination, especially when the bacterial strains are closely associated with the seeds (Novak et al., 2014). Interestingly, even strains producing lower amounts of IAA demonstrate a continuous release, leading to improved plant growth. This underscores the potential utility of IAA-producing strains in enhancing the efficiency of orchid propagation methods.

#### 4.1.2 Production of ethylene to enhance the stress tolerance of plants

PGPB not only enhances plant growth but also helps mitigate stress-induced damage. Under biotic and abiotic stress, plants often overproduce ethylene, which inhibits root elongation and development. Certain endophytic bacteria possess the enzyme ACC deaminase, which breaks down ACC, the ethylene precursor, into *α*-ketobutyrate and ammonia. By utilizing ACC as a nitrogen source, these bacteria reduce ethylene levels, alleviating stress-related growth inhibition.

Endophytic bacteria were isolated from three species of Mediterranean terrestrial orchids: *Spiranthes spiralis, Serapias vomeracea*, and *Neottia ovata*. Taxonomic identification, based on the 16S rRNA gene, revealed bacterial isolates belonging to the genera *Pseudomonas, Pantoea, Rahnella, Staphylococcus, Sphingomonas, Microbacterium, Streptomyces, Fictibacillus and Bacillus*. These isolates underwent various assays to elucidate their potential beneficial functions as PGPB as well as to assess their salinity and drought tolerance, and interactions with other components of the orchid microbiota. Among the bacterial endophytes, those producing ACC deaminase demonstrated the ability to grow on culture media containing NaCl (approximately 50% of all isolates) and 10% PEG (all tested isolates), indicating their capacity to tolerate abiotic stress such as salinity and osmotic stress. The adaptability of these bacterial endophytes to salinity and osmotic stress raises questions about whether this trait is a direct response to the Mediterranean climate. Further investigation is needed to discern whether these adaptations serve an additional role as abiotic stress alleviators for the host orchid plants, contributing to their resilience in challenging environmental conditions (Alibrandi et al., 2021).

The above findings presented are reinforced by the examination of rhizospheric bacteria from various orchid species, including *Anacamptis pyramidalis, Himantoglossum caprinum, Limodorum abortivum, Platanthera bifolia, Serapias vomeracea* subsp. *laxiflora, Spiranthes spiralis, Ophrys apifera, Ophrys sphegodes, Orchis coriophora, Orchis laxiflora, Orchis provincialis, and Orchis tridentata*. These bacteria were systematically screened for diverse plant growth-promoting traits, encompassing phosphate solubilization, ACC deaminase activity, and IAA production (Altinkaynak and Ozkoc, 2020).

### 4.2 Nutrient mobilization by plant growth promoting bacteria in orchid growth

Endophytic and rhizospheric bacteria play a crucial role in supporting the availability and absorption of essential plant nutrients, including nitrogen, iron, and phosphorus. These beneficial bacteria contribute to nutrient cycling and enhance the overall nutrient uptake efficiency of plants, fostering healthier growth and development. Their symbiotic interactions with plant roots facilitate the acquisition of limiting nutrients, thereby playing a vital role in optimizing plant nutrient utilization in diverse ecosystems.

#### 4.2.1 Nitrogen availability

Bacteria can enhance plant nitrogen availability by fixing atmospheric nitrogen through the activity of the conserved enzyme nitrogenase. Nitrogen-fixing isolates from the aerial roots of Rhynchostylis retusa, including Achromobacter, Arthrobacter, Brevibacterium, Klebsiella, Mixta, Bacillus, Pantoea, and Pseudomonas, demonstrated nitrogenase activity via acetylene reduction assays. This ability was further validated through PCR amplification of the nifH gene and detection of a 37 kDa nitrogenase reductase enzyme band. Increased total carbon and nitrogen levels in leaves, stems, and roots suggest enhanced nitrogen accumulation, primarily driven by bacterial nitrogen fixation in the roots (Yadav et al., 2023).

The biological nitrogen fixation ability of endophytes, including *Paenibacillus taichungensis, Enterobacter* sp.*, Rhizobium* sp., *Paenibacillus* sp., *Pseudomonas* sp., *and Paenibacillus pabuli,* were determined in various environments. Despite all isolates exhibited nitrogen fixation ability, there were variations in their fixation capacities across different culture media, with more efficient nitrogen-fixing isolates obtained from their natural habitat. Upon inoculation of these growth-promoting bacteria *in C. walkeriana* plants, a notable increase in growth parameters, including leaf area, number of roots, root length, and plant height, was observed, indicating a positive impact on the plant’s overall development (Andrade et al., 2023).

#### 4.2.2 Phosphorus availability

Phosphorus in soil often forms insoluble complexes, rendering it largely unavailable to plants. While chemical phosphate fertilizers are widely used, they are costly, environmentally harmful, and inefficient, with up to 75% of phosphorus remaining inaccessible. Utilizing microorganisms as biofertilizers offers a sustainable alternative by enhancing phosphorus availability and reducing dependence on chemical inputs. Endophytic bacteria improve phosphorus solubilization through mechanisms such as acidification, chelation, ion exchange, and organic acid production. Advances in microbial molecular engineering further support eco-friendly and efficient agricultural practices.

*Herbaspirillum frisingense* and *Stenotrophomonas maltophilia,* isolated from the roots and leaves of *Cymbidium* sp., exhibit phosphorus solubilization capabilities and other plant growth-promoting traits. Inoculating orchid plantlets with these bacteria resulted in viable growth, as evidenced by various growth parameters (Gontijo et al., 2018). These findings find support in another study, where *Collimonas pratensis* and *Chryseobacterium* sp. bacterial isolates demonstrated the ability to solubilize phosphate. These isolates exhibited a higher phosphorus solubilization index, coupled with additional plant growth-promoting traits. The collective evidence underscores the potential of these bacteria not only in phosphate solubilization, but also in contributing to overall plant growth and development through various PGP mechanisms (Herrera et al., 2020b). The study on meristem endophytes of *Cymbidium eburneum* revelead presence of only one genus *Paenibacillus*, with two species *P. lentimorbus* and *P. macerans*. These two strains show higher values for phosphorus solubilization index and promote plant growth under greenhouse conditions. They significantly enhance the biomass in shoots and roots of orchid seedlings (Faria et al., 2013).

These findings underscore the potential of integrating *in vitro* propagation with bacterial inoculation as an effective strategy to boost orchid growth, lower production costs, and improve nutrient uptake. In particular, the use of diazotrophic bacteria shows promise in accelerating plant acclimatization, offering a valuable edge to the floriculture and ornamental plant industry in terms of both productivity and market competitiveness. Beyond horticulture, this biotechnological approach holds broader agricultural significance, with the ability to reduce reliance on chemical fertilizers and deliver substantial economic and environmental benefits.

#### 4.2.3 Iron availability

Iron is essential for plant metabolism and chlorophyll synthesis, and siderophores produced by microbes and plants enhance iron uptake while also aiding heavy metal mobilization and environmental remediation. Siderophores, produced by bacteria, fungi, and plants, are iron scavengers gaining attention for environmental bioremediation. In soils, abundant iron exists as crystalline and amorphous oxides, with microbial siderophores aiding in iron acquisition under Fe-limited conditions (Ma et al., 2016). The ability of these chelators to alter dye colours on agar plates indicates their role in iron acquisition by bacteria. Additionally, siderophores can mediate the production of reactive oxygen species, promoting the biodegradation of organic contaminants.

Endophytic bacteria isolated from the pods of two hybrids of Vanilla orchids, including *Bacillus thuringiensis, Bacillus inaquosorum, Bacillus subtilis, Bacillus siamensis, and Pseudomonas fluorescens,* demonstrated elevated siderophore production, as well as the production of IAA and phosphorus solubilization. The research highlighted a correlation between bacterial diversity and the host genotype. The isolated bacteria show potential for Phyto stimulation, and further testing on model plants is recommended to assess their effects on plant growth. This suggests a potential application of these bacteria in enhancing plant growth through beneficial interactions (Lalanne-Tisne et al., 2023).

### 4.3 Indirect growth promotion by suppression of phytopathogens

Endophytic bacteria can also enhance host plant growth indirectly by enhancing their defense mechanism against phytopathogens and plant pests through the production of various substances such as antibodies, toxins, siderophores, hydrolytic enzymes, antimicrobial and antifungal organic compounds (Woźniak et al., 2019). These bacteria exhibit antagonistic effects against phytopathogens. This antagonism creates a protective environment for the host plant, promoting its overall health and growth by suppressing the harmful activities of potential pathogens and pests. *Actinobacteria, Bacillus, Enterobacteor, Paenibacillus, Pseudomonas* and *Serratia* are the most reported genera for their antimicrobial activity against phytopathogens.

*Bacillus spartinae*, isolated from the roots of *Cymbidium* orchids, was investigated for its antibacterial and antifungal activities against *Bacillus cereus, Staphylococcus aureus, Pseudomonas aeruginosa, Escherichia coli, Ganoderma boninense, Pythium ultimum, and Fusarium solani*. Intriguingly, the antibacterial and antifungal activities of the fractions contradicted those of the crude extracts, suggesting complex interactions among compounds in the fractions (Chua et al., 2023). Future research should focus on further purifying antibacterial fractions to determine their specific potency, testing with a broader concentration range and various pathogens, and conducting cytotoxicity assessments for potential applications in treating human or animal-related bacterial pathogens.

Endophytic bacteria isolated from the stems of *Dendrobium* were dominated by *Bacillus* and *Lysinibacillus* genera. *The antimicrobial* activities of isolated endophytic bacteria were evaluated against phytopathogens, *A. rolfsii*, *M. roridum*,and *P. carotovorum* subsp. *Actinidiae* which can cause southern blight, tar spot, and soft rot disease, respectively. Five different genera of endophytes showed antimicrobial activity against at least two phytopathogens, namely *Paracoccus*, *Pseudomonas*, *Microbacterium*, *B. subtilis*, and *Streptomyces.* It is evident from past studies that *Pseudomonas* is known for its beneficial interaction with its host plant, enhancing systemic resistance, promoting growth, suppressing pathogens, and so on. In this study, the genus *Pseudomonas* could not only produce Dendrobine-Type Sesquiterpenoid Alkaloids but also enable effective antagonize the phytopathogens of *Dendrobium* (Wang et al., 2022). These findings shed light on the intricate relationship between endophytes and host plant growth and microbial-plant interaction, offering valuable insights for future research on the underlying mechanisms.

*Streptomyces* sp. isolated from various orchids, including *Dendrobium nobile, Dendrobium chrysotoxum, Dendrobium moschatum*, and others, underwent screening for several PGP traits, extracellular enzyme production, and antifungal activity. The endophytes exhibited the production of extracellular enzymes such as chitinase, cellulase, pectinase, and protease, contributing to organic matter decomposition and plant growth stimulation while preventing diseases by inhibiting soil-borne pathogens. The *Streptomyces* sp. isolates were further evaluated for their antifungal activity against 10 fungal phytopathogens, demonstrating antagonism against pathogens like *Fusarium oxysporum*, *Rhizoctonia solani* and others. Screening for the chitinase gene among these isolates, which exhibited antifungal and chitinase activity, indicated the presence of unique mechanisms to hinder fungal phytopathogen growth. However, the involvement of additional mechanisms or bioactive substances in the observed antifungal activity suggests that the presence or absence of chitinase genes alone does not exclusively determine the isolates’ antagonistic activity (Saikia et al., 2022).

PGPB also activates plant defense mechanisms, leading to systemic protection against plant pathogens. This phenomenon is referred to as Induced Systemic Resistance (ISR). ISR is a phenomenon where plants are primed to enhance their defense mechanisms, providing protection to unexposed plant parts against potential future attacks by microbes and herbivorous insects. Endophytic bacteria play a crucial role in initiating ISR through the activation of signaling pathways, such as those mediated by salicylic acid (SA), jasmonic acid (JA), and ethylene (ET). These pathways constitute a network of interconnected signaling cascades that collectively contribute to the induction of ISR. By triggering these defense pathways, endophytic bacteria empower plants to mount a more robust and efficient response to subsequent pathogenic challenges, demonstrating the intricate and interconnected nature of plant-microbe interactions in bolstering plant defense mechanisms (Okazaki et al., 2013).

In the context of ISR by plant-associated bacteria, initial demonstrations involved *Pseudomonas* spp. and other gram-negative bacteria. Summarized findings from various published studies highlight specific strains of Bacillus species, such as *B. amyloliquefaciens, B. cereus, B. pasteurii, B. pumilus, B. mycoides, B. subtilis* and *B. sphaericus*, highlighting significant reductions in the incidence or severity of diverse diseases across various hosts. Greenhouse and field trials have validated the ability of these strains to elicit ISR. Protection conferred by ISR induced by *Bacillus* spp. has been observed against leaf-spotting fungal and bacterial pathogens, systemic viruses, crown-rotting fungal pathogens, root-knot nematodes, stem-blight fungal pathogens, damping-off, blue mold, and late blight diseases. Moreover, *Bacillus* spp. exhibiting ISR induction also tends to promote plant growth. Mechanistic insights suggest that ISR elicited by *Bacillus* spp. is linked to ultrastructural changes in plants during pathogen attacks and associated cytochemical alterations (Kloepper et al., 2007).

*Pseudomonas aeruginosa* has been shown to produce metabolites with strong metal-chelating properties and moderate reducing power, contributing significantly to improved cold tolerance in *Phalaenopsis* orchids. This enhancement is evidenced by reduced malondialdehyde (MDA) levels and decreased electrolyte leakage. The bacterium also conferred increased resistance to soft rot disease caused by *Erwinia chrysanthemi*. Protein analysis revealed elevated levels of antioxidant enzymes, including catalase (CAT) and ascorbate peroxidase (APX), as well as pathogenesis-related (PR) proteins and lipoxygenase 1 (LOX1). Transcriptomic analysis of strain Y1M indicated its influence on gene expression related to the iron-deficiency response (mediated by miRNA), regulation of reactive oxygen species (ROS) homeostasis, and jasmonic acid (JA) biosynthesis and signaling. Additionally, transcription factors linked to cold stress response, such as C-repeat binding factor 1 (CBF1), and those associated with cell wall strengthening, like MYB26, showed increased expression. These results confirm the activation of key cellular pathways involved in stress tolerance, revealing overlapping mechanisms for both abiotic (cold) and biotic (pathogen) stress responses (Chuang et al., 2022).

**Figure 3:**
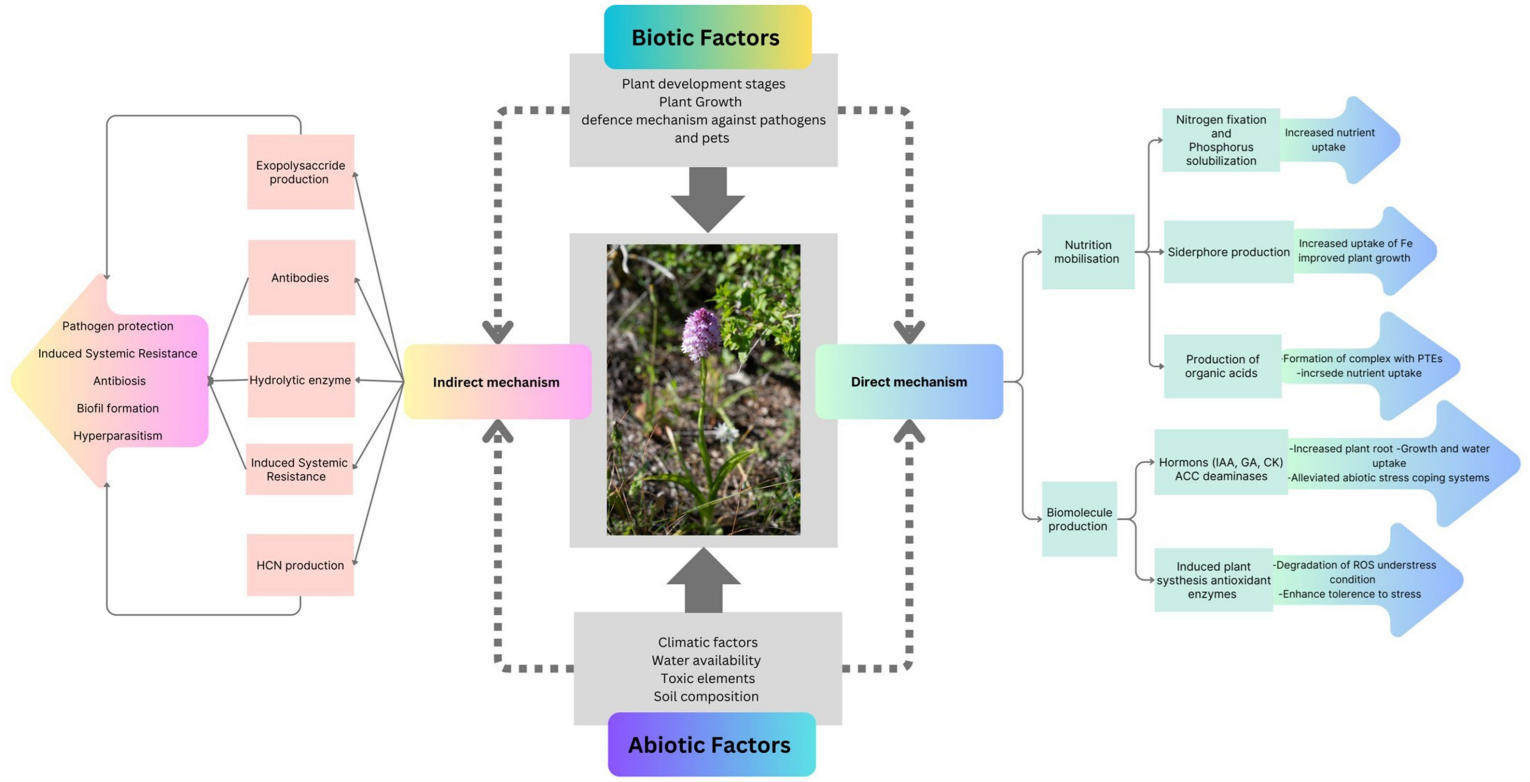
The modes of action for plant growth-promoting bacteria (PGPB) encompass both direct and indirect mechanisms. These mechanisms include nutrient mobilization, biomolecule production, enzyme and antibody functions, and the induction of systemic resistance (IAA, Indole acetic acid; GA, Gibberellic acid; CK, Cytokinin; ACC, 1-aminocyclopropane-1-carboxylate deaminase).

Figure 3 and Table 3 summarize key PGPB associated with orchids, detailing their specific functions and contributions to orchid growth, development, and stress resilience. This compilation supports the discussion on the functional diversity and significance of these PGPB in supporting orchid health and development.

## 5 Summary of key findings

Firstly, this review highlighted the diversity and beneficial role of PGPB for the orchids. The diverse benefits mentioned suggest that PGPB can play a crucial role in enhancing orchids growth and disease control. This review identifies specific attributes and behavior traits of PGPB that can be effectively utilized at the different life cycle stages (seed, protocorm, plantlet and mature plant).

Secondly, this review aimed not to list specific PGPB traits but to highlight key ecological and functional characteristics essential for understanding symbiotic associations, which are crucial for the effective application of PGPB in the field.

Thirdly, modern molecular biology tools, especially ‘omics’ technologies, offer powerful means to explore and distinguish the genetic and metabolic traits involved in plant–PGPB interactions, aiding in the identification of phytopathogens, endophytes, and other microbes. This increased precision allows for a clear distinction between mutualistic microbes and pathogens, acknowledging that the boundaries between these groups may not always be straightforward from an ecological perspective.

Fourthly, the selected PGPB possess one or more effective mechanisms of action, such as mobilizable bioactive compounds for biocontrol. Importantly, the antagonistic properties of the PGPB must exhibit restricted bioactivity to avoid influencing beneficial non-target species like pollinators (e.g., honeybees and bumblebees) and earthworms.

## 6 Gaps in current knowledge and future research directions

**Figure 4:**
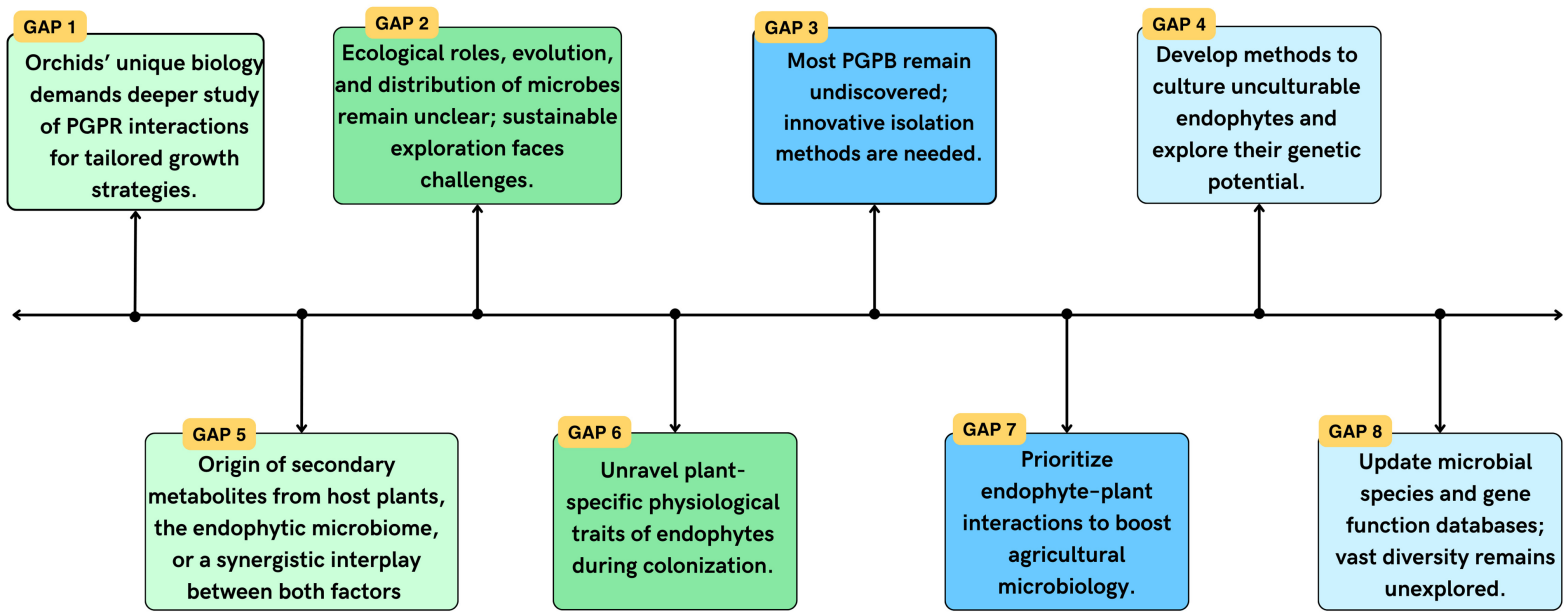
Gaps in current knowledge and future research directions.

Our current understanding of orchid-related PGPB is limited, both in terms of diversity and functional activities. The unique biology of orchids, particularly their specific mycorrhizal associations and nutrient requirements, imposes constraints on identifying suitable PGPR strains for orchid-microbial biotechnology. The scarcity of knowledge in this field hinders the exploration of the full potential of PGPR in enhancing orchid growth and health. Addressing these gaps (Figure 4) through further research is essential to unlock the possibilities of utilizing PGPRs effectively in orchid cultivation and microbial biotechnology.

## 7 Concluding remarks and prospective

The bacterial endophytes found in orchids play a crucial role in their germination and growth across different developmental stages. Traditionally, orchid mycorrhizal fungi have been recognized as essential symbionts for orchid development. However, recent research indicates that endophytic and rhizospheric bacteria can serve as effective alternatives to fungi. These PGPB are pivotal in various aspects such as nutrient uptake, biomass production, bolstering resilience against environmental stress, and fortifying defense mechanisms against pathogens. Numerous studies have underscored their potential in replacing harmful chemicals like fertilizers, fungicides, and pesticides, thus promoting eco-friendly agricultural practices without disrupting conventional farming methods. Moreover, these bacteria have the capability to enhance soil health by augmenting the availability of natural nutrients. Consequently, there is a pressing need for further investigation into microbial inoculants in degraded soils and their interactions with plants and native microbial communities. We advocate for collaborative efforts among global taxonomists, ecologists, natural product chemists, agronomists, and bioengineers to effectively harness the biodiversity and biotechnological potential of PGPB. A multidisciplinary approach is essential for comprehensive exploration and utilization of these microorganisms in various fields.
